# Motivation, self-efficacy, and identity—double-edged swords for relapse prevention in patients with alcohol related cirrhosis

**DOI:** 10.1093/alcalc/agaf027

**Published:** 2025-05-22

**Authors:** Christopher Oldroyd, Tamar Avades, Graham P Martin, Caitlin Notley, Michael E D Allison

**Affiliations:** University of Cambridge, Cambridge University Hospitals NHS Foundation Trust, Cambridge Liver Unit, Addenbrooke’s Hospital, Hills Rd, Cambridge, Cambridgeshire, CB2 0QQ, United Kingdom; Hepatology Research Group, University of Plymouth, Plymouth, Devon PL4 8AA, United Kingdom; The Healthcare Improvement Studies Institute, University of Cambridge, Strangeways Research Laboratory, 2 Worts’ Causeway, Cambridge, Cambridgeshire, CB1 8RN, United Kingdom; Addiction Research Group, University of East Anglia, Norwich Research Park, Norwich, Norfolk NR4 7TJ; Cambridge University Hospitals NHS Foundation Trust, Cambridge Liver Unit, Addenbrooke’s Hospital, Hills Rd, Cambridge, Cambridgeshire CB2 0QQ, United Kingdom; Cambridge NIHR Biomedical Research Centre, Hills Rd, Cambridge, Cambridgeshire, United Kingdom

**Keywords:** cirrhosis, hepatitis, relapse, abstinence, alcohol

## Abstract

**Background and Aims:**

Despite the critical importance of alcohol abstinence for patients with advanced liver disease, rates of returning to alcohol remain high and engagement with relapse prevention interventions is low. This study explores the potential barriers to relapse prevention in these patients.

**Methods:**

Semi-structured interviews were conducted with patients who had alcohol-related cirrhosis or alcohol-associated hepatitis. Interviews took place during a hospital admission. The study methodology was informed by a constructivist grounded theory approach.

**Results:**

Thirty-three participants were recruited from two sites. Participants had a mean age of 52 (range 30–60) and there were 10 female participants (30%). Most participants were actively drinking alcohol at time of admission (*n* = 26) and 16 participants were interviewed during their index admission with alcohol-related liver disease.

A renewed understanding of the health risk posed by future alcohol made participants confident that they would not return to alcohol use and participants felt that the most important factor in relapse prevention was their own motivation and willpower. However, many rejected the identity label of ‘alcoholic’ and drew a distinction between themselves and ‘bad drinkers’. These factors combined to create a barrier to relapse prevention therapies, since participants felt these were neither appropriate nor necessary for them.

**Conclusions:**

Enhanced self-efficacy, a belief in the importance of willpower, and a rejection of the alcoholic identity can together act to reduce engagement in relapse prevention in patients with advanced liver disease. Relapse prevention interventions should be reframed or redesigned to address these barriers.

## Teaser

**Short Summary:** For patients with alcohol related cirrhosis, abstinence is the key determinant of outcome. Through interviews with patients during an admission to hospital we identified that enhanced self-efficacy, a belief in the importance of willpower, and a rejection of the alcoholic identity may act together to reduce engagement in relapse prevention.

## Introduction

Alcohol-related liver disease (ArLD) describes damage done to the liver as a result of harmful consumption of alcohol. There is a spectrum of disease severity with the most severe consequences being alcohol-associated hepatitis and/or alcohol-related cirrhosis (Seitz *et al*. 2018). These conditions account for the greatest burden of hospitalisations, liver transplantation, and mortality from liver disease in Europe and the USA (Devarbhavi *et al*. 2023). In England, there has been a 74% rise in premature deaths from ArLD over the last 20 years (Office for Health Improvement and Disparities 2023). Abstinence from alcohol provides a 4-fold mortality benefit at 2 months in patients with alcohol-associated hepatitis (Potts *et al*. 2013) and doubles survival at 36 months in patients with alcohol-related cirrhosis and portal hypertension (Hofer *et al*. 2023).

Despite this, rates of returning to alcohol use amongst patients with advanced ArLD are high (Thursz *et al*. 2015, Lim *et al*. 2024). The term ‘relapse prevention’ encompasses any intervention, which is aimed at preventing a return to harmful drinking. These include, but are not limited to, psychological and pharmacological treatments for alcohol use disorder (AUD). In practical terms, relapse prevention work often draws on multiple modalities and involves health care practitioners and patients working together to minimise the risk of return to alcohol. Engagement with such therapies by patients with ArLD has been associated not only with reduced risk of returning to alcohol (Gratacós-Ginès *et al*. 2024), but also with decreased rates of hepatic decompensation (Rogal *et al*. 2020, Oldroyd *et al*. 2023). However, uptake of and engagement with relapse prevention after a diagnosis of alcohol-related cirrhosis is as low as 10%–15% (Mellinger *et al*. 2019, Rogal *et al*. 2020). It is vital to understand why it is so common for patients with advanced liver disease to return to harmful drinking and why engagement with relapse prevention is so poor.

Hospital admission presents an opportunity for relapse prevention for at least three reasons. First, it might act as a ‘teachable moment’ (Lawson and Flocke 2009, Clark and Moss 2011)—a point at which behaviour change might be embedded. Second, during admission patients experience an enforced period of abstinence (often with medically assisted alcohol withdrawal), potentially enhancing their ability to remain abstinent following discharge. Finally, patients may have access to specialist services to support them in future abstinence.

The aim of this study was to explore the views and experiences of relapse prevention in patients admitted to hospital with alcohol-related cirrhosis.

Qualitative studies allow us to explore phenomena in depth, providing insights that might be missed by surveys or analysis of quantitative data. Such studies in patients with alcohol-related cirrhosis are lacking and the need for further research in this area has been highlighted (Singal *et al*. 2021, Horrell *et al*. 2022). Much existing qualitative work examining return to alcohol in patients with liver disease considers patients who are being considered for or have received a liver transplant (Heyes *et al*. 2016, Hochheimer *et al*. 2019). This population is by definition distinct from the majority of patients with ArLD. Transplant consideration is contingent on a period of abstinence and liver transplant recipients will have already been identified as being at low risk of returning to harmful drinking. Mellinger *et al*. have conducted two qualitative studies examining alcohol use in patients with cirrhosis but both recruited patients from outpatient clinics (Mellinger *et al*. 2018, Mellinger *et al*. 2024).

One single-centre qualitative study has recently explored abstinence and relapse prevention amongst hospitalised patients with alcohol-related cirrhosis (Hemrage *et al*. 2024). Hemrage at al. interviewed patients who were already participating in a clinical trial of contingency management. These patients had therefore already exhibited a willingness to engage in an intervention for AUD, which may have influenced the findings. All of the aforementioned studies applied predefined theoretical frameworks to their analysis. This study adopted an inductive approach based on grounded theory in the hope of challenging existing theory and generating novel insights.

## Methods

We conducted semistructured interviews with patients with alcohol-related cirrhosis and/or alcohol-associated hepatitis during a hospital admission. We included patients who were drinking alcohol at the point of admission and those who had achieved abstinence. We excluded patients who had received a liver transplant. There were no age restrictions. To encourage participation, patients were offered a £25 voucher. Participants were required to have capacity to provide informed consent and participate in the interview in English. The first author used clinical judgement to ensure participants were well enough to participate in the study. We used purposive sampling with a strategy of maximum variation. We sought to achieve variation in age, gender, and social status. We also sought variation in the narratives surrounding both liver disease and alcohol use. Specifically, we hoped to recruit a spectrum of patients encompassing those with a new diagnosis of liver disease without previous contact with alcohol services, through to patients with numerous previous admissions with several previous attempts to remain abstinent. We included patients who had achieved and maintained abstinence since it was considered that important knowledge could be gained from their experiences. The goal was to recruit a sample that reflected the wider population of patients with advanced ArLD. Details of patients who were approached for the study but did not consent, were unavailable or were ineligible, were not collected. Recruitment took place from two centres in the UK—a liver transplant unit that admits patients directly and takes regional referrals and transfers, and a district general hospital in an area of disproportionately high ArLD deaths.

Interviews were conducted by the first author who was also the principal analyst. The interviews all took place in person and were audio recorded. Recordings were transcribed verbatim by a professional transcription service with any information, which might clearly identify the participant removed. Transcripts were stored and analysed on NVivo V20.7.2. Most interviews took place in a hospital, usually in a private room; some participants declined this option and were interviewed at the bedside. One interview took place at the participant’s home within 2 days of discharge. Participants had the option to be accompanied by a friend or relative. Interviews were semistructured and used a topic guide, which evolved iteratively throughout the study.

A patient and public advisory group of six members with lived experience of alcohol-related cirrhosis and AUD was advised on this study. They provided feedback on the methods and reviewed study-related documents (consent forms, participant information sheets, and study guide). Evolving themes were discussed with the patient and public advisory group and insights from those discussions used to influence the study guide and subsequent interviews.

### Analysis

The study methodology was informed by a constructivist grounded theory approach (Charmaz 2006). Grounded theory is a well-established method in qualitative healthcare research (Chapman *et al*. 2015) and is particularly relevant to this topic where there is a paucity of relevant qualitative data. It is an inductive method of developing theory based on the collected data. The literature was consulted as new themes were derived from the data. Data collection and analysis occurred concurrently. Interview transcripts were initially coded line by line. These codes were then reviewed and combined into wider themes or codes were created or removed. To establish the overall narrative and key recurring themes, each transcript was further reviewed and audio recordings were listened to in full. As data collection progressed, efforts were made to identify and recruit participants who might have characteristics or experiences that would address gaps in the evolving grounded theory. Recruitment stopped when analysis of new interviews resulted in the identification and elaboration of no new codes or themes, a point known as theoretical saturation.

Data and coding were regularly discussed with co-authors and with the patient advisory group. A random sample of transcripts was sent to a second researcher (TA) who independently coded these. Codes and key themes from these transcripts were then compared and any differences were resolved by discussion and mutual agreement. In reporting the findings, pseudonyms are used to represent individual respondents and their quotations.

### Ethics

 Ethical approval was received from the NHS Health Research Authority, Cornwall and Plymouth Research Ethics Committee (REC reference 22/SW/0077).

## Results

Between March 2023 and April 2024, 33 participants were interviewed for the study. Relevant sample characteristics are summarised in Table 1. The cohort achieved variation in age and gender, which mirrored the distribution of these characteristics seen in the overall cohort of patients with ArLD cirrhosis in the UK (Office for National Statistics 2022). Variation was also achieved in presenting symptoms, liver disease severity, history of previous admissions, and histories of alcohol use, which is explored below. While there was an even distribution of participants across deprivation deciles, this did not reflect the wider population of patients with ArLD cirrhosis in the UK where hospital admissions and mortality are significantly skewed towards patients from areas of high deprivation (Office for Health Improvement and Disparities 2024).

### Motivation, confidence, and willpower

Almost every participant interviewed spoke of the need to reduce their alcohol intake or become completely abstinent after discharge. The threat posed by the development of tangible physical consequences and subsequent hospitalisation provided clear motivation to change. Participants frequently described the desire to avoid further hospital admissions and prevent additional deterioration of their physical health. They clearly recognised that the consequences of future alcohol use could be fatal.

Oscar (52 years old)—‘I’m okay with knowing that I have to stop but I would have carried on. I would have carried on, if I hadn’t have turned yellow, yes.’

Clare (53 years old)—‘After what I’ve been through in here and I don’t think my body would take it again. I just don’t think my body will take it.’

Luke(48 years old)—‘This time has to be different. If I keep on going down this route, I’m going to die.’

Many also wanted to preserve their health (and their sobriety) for the sake of family or friends.

Mark (59 years old)*—*‘And why do you need to stop? It’s because you’re going to ruin your life; it’s not because you’re going to be ill. It’s the broken relationships.’

Caring responsibilities and desire to see children or grandchildren were commonly cited as motivation to remain abstinent. The impact of alcohol intake on others was in some cases a greater motivator to remain abstinent than the impact on a participant’s own health.

**Table 1:** Sample demographics.

Demographics	*n* = 33
Age (mean)	52 (range 30–69)
Gender Female Male	10 (30%) 23 (70%)
Ethnicity White Born in UK	33 (100%) 30 (91%) (Poland, 2; Russia,1)
Deprivation deciles 1–2 (most deprived) 3–4 5–6 7–8 9–10 (least deprived)	4 (12%) 7 (21%) 8 (24%) 8 (24%) 6 (18%)
Admission characteristics Transplant Centre (local patients) Transplant Centre (transferred patients) District General Hospital Admissions in previous 5 years (mean) Participants with 10+ admissions Drinking at admission Index admission with liver disease	20 (61%) 5 (15%) 8 (24%) 5.4 (range 0–45) 7 (21%) 26 (79%) 16 (48%)
Primary reason for admission Decompensated cirrhosis Ascites Alcohol associated hepatitis Variceal bleeding Encephalopathy Other (e.g. alcohol withdrawal syndrome)	29 14 11 3 1 4
Liver disease scores Median MELD Median UKELD	17 (range 6–37) 55 (range 46–75
Selected co-morbidities Depression Anxiety Other substance use Epilepsy/seizures Post traumatic stress disorder Bipolar disorder	9 6 7 5 1 1

MELD - Model of End-Stage Liver Disease. UKELD - United Kingdom Model for End-Stage Liver Disease.

In the face of an immediate risk to physical health and therefore enhanced motivation, participants expressed high levels of confidence in their ability to maintain abstinence after discharge. Even participants who had returned to harmful alcohol use on multiple occasions following life-threatening hospital admissions often vowed that this time would be different.

Jonathon (49 years old)—(on relapse prevention) ‘I don’t think—and it’s not going to be challenging to me at all. When I put my mind to something, I know I can do it.’

Linked to a renewed motivation and confidence was the belief that future abstinence was primarily dependent on willpower. Participants described the need to take ownership of their own relapse prevention and saw success as dependent on complete commitment. Individual determination and resolve was seen as the most important (or even the only) factor that determined risk of relapse.

David (60 years old)—‘I’d like to think that I could achieve it myself, yes, I am determined, you know, I don’t want to go through this anymore, you know.’

Matthew (57 years old)—‘You can have all the support you want in the world but the only person that’s going to stop you drinking is yourself. You know, you’ve got to make a decision.’

### Alcohol histories and identity

Participants could be broadly divided into two groups based on their self-described relationship with alcohol use. One, smaller, group of respondents clearly described both physical and psychological substance dependence. Often, they had struggled with this for many years and had several unsuccessful attempts to stop drinking. These participants described histories of trauma, abuse, relationship breakdown, and struggles with mental health. Some were insightful about having a dependency on alcohol.

Thomas (55 years old)—‘But what I found is when I’ve tried to cut back or moderate, once I have one I can’t stop. It’s sort of uncontrollable. It’s controlling me rather than me controlling it.’

However, most participants rejected the label of ‘alcoholic’. Until their diagnosis of liver disease, many had not faced significant adverse consequences of alcohol use and therefore had not viewed it as problematic. Participants understood that they had drunk above recommended limits, resulting in severe liver damage, but did not see this as equating to alcohol dependency. A distinction was frequently drawn between their own drinking patterns and so called ‘bad drinkers’:

Jennifer (36 years old)—‘It’s not like I’m a bad drinker. I drink for like relaxation, so it would just be with my friend, sit there and have a laugh and stuff. It’s not like we’re going to go out and be like hooligans or something like that. Or I’m making like risky choices, things like that.’

Oliver (43 years old)—‘But the one thing I will say—and it’s gospel—is I’ve never been what I’d put down to be (an alcoholic)—I’ve never drank in the morning; I’ve never finished off a bottle of whisky.’

Participants frequently drew moral boundaries between acceptable and unacceptable alcohol intake. For example, while all participants had consumed alcohol daily, most stated that they did not let this affect their work, and that they would never drink and drive. Many participants reported that while they were aware of the consequences of harmful alcohol use, they did not perceive it as a risk to them individually. For some participants, reassuring test results (such as blood tests) further supported this position. Recommended guidelines were not viewed as relevant to every individual.

Jonathon (49 years old)—‘I thought, you know, my liver will be fine. My dad’s a real heavier drinker than me and he’s telling me, oh, he’s fine. Other people I know are heavier drinkers, they’re fine. I thought, you know, I’ll be fine.’

Instead, social norms and pressures appeared to be the key driving influences in participants’ histories of harmful alcohol use. Social context normalised patterns of harmful drinking. For some participants, alcohol use gradually escalated from harmful drinking, which was in keeping with specific social norms, to more significant alcohol dependency. Many participants, however, insisted that their alcohol use was no different from that of their peers, even up to the point of a diagnosis of severe ArLD. Several participants described a culture of alcohol intake associated with their employment. The same social groups and pressures were then seen to present a risk of relapse after discharge.

Finlay (60 years old)—‘It was with friends, like builders, we all used to meet in the pub every night and we’d drink loads and loads. When you’re amongst all those people, you don’t think you have a problem because everyone else is doing what you do.’

Beyond social pressures, participants described the specific personal role that alcohol played in their lives. They used alcohol to cope with stress, anxiety, and insomnia. Many participants described escalating alcohol use in response to past or present trauma and as a way of dealing with major life events such as relationship breakdown and job loss. Boredom, inactivity, and isolation were also frequently cited as reasons for ongoing alcohol use and relapse to alcohol after a previous hospital admission. Boredom and isolation could lead to drinking at home to pass the time or going to the pub for social contact.

James (43 years old)—‘When I moved in on my own it became a lonely place and it was a case of put the TV on, walk the dog, and come home, and open a bottle of wine.’

In keeping with the theme of boredom and isolation, several participants traced their increased drinking to the COVID pandemic and associated lockdowns. Common to all such identified causes, though, was their external origin. Since they constructed their own drinking behaviour as driven by social and environmental stimuli, and saw ‘alcoholism’ in terms of individual pathology, participants could characterise their own alcohol consumption as something other than alcohol dependency.

### Relapse prevention

Patients felt motivated to remain abstinent post discharge and expressed confidence in their ability to sustain abstinence without an additional support. In this context, and given the perceived importance of willpower in preventing relapse, many rejected the offer of formal relapse prevention interventions. Negative perceptions of ‘bad’ drinkers also translated into negative attitudes towards alcohol services. Some participants discussed service users in stigmatising ways:

Finlay (60 years old)—‘They were like drug addicts. I didn’t want to sit in there with them, so I just about turned and walked out.’

Some participants felt that talking about alcohol would only increase their risk of returning to harmful drinking.

Anthony (60 years old)—‘But what am I going to talk to them about what I haven’t already done? I also feel every time I go there, I’m just bringing it back up again.’

More positive experiences tended to be described by participants who had formed a personal connection with a consistent member of the relapse prevention service. With regard to pharmacotherapies for relapse prevention, many participants reported having limited or no knowledge of these medicines. While there were individual stories of success with pharmacotherapies, most reported they had not been offered these treatments but that they were willing to consider using them. A few participants had used apps and other online resources to support their abstinence.

Rather than traditional relapse prevention therapies, participants who had achieved periods of abstinence had found new ways of coping with triggers and/or of replacing alcohol with a substitute. This might be through hobbies, activities, or alternative (nonalcoholic) drinks. Those planning for discharge recognised that keeping busy was an important protection against returning to harmful drinking.

John (30 years old)—‘Well probably the hardest part of it will be finding activities to replace, or to fill the time when I was drinking, or find other ways to sort of lift myself up, as in, in the mood. So something to do.’

We specifically asked participants about the use of no- or low-alcohol (NoLo) products when this was not spontaneously raised. NoLo products are beverages that are made to have either significantly reduced or no alcohol content, while still replicating the taste and experience of standard alcoholic drinks. Opinions were divided on the value of these products, with many participants concerned that using them would be a pathway back to harmful use of alcohol. Others viewed them as a potentially useful replacement with the main potential benefit to be found at social events or special occasions where friends and relatives would be consuming alcohol.

Participants also highlighted the need to make changes to their previous environments. Common strategies included removing alcohol from the home, avoiding pubs, and handing over or destroying credit cards.

Peter (50 years old)—‘What I’m going to do is my credit card I’m going to give to my parents, so I’ve got no money to actually buy alcohol.’

For some participants, alcohol consumption had been tied to employment (either through a culture of drinking or because of work pressures). Those participants all recognised the need to find alternative employment or stop working completely. A few described their intention to pursue charitable work, which would not only fill their time, but provide a sense of purpose and boost self-esteem. Some had supportive family members with whom they could live for a period to help them avoid alcohol.

## Discussion

This study presents an exploratory investigation of the views and experiences of relapse prevention after hospital admission in participants with advanced ArLD. Participants understood the risk of future drinking, which had been amplified by the development of tangible health consequences and hospitalisation. However, this enhanced perception of risk led to disproportionately high levels of motivation, and confidence amongst participants in their ability to remain abstinent. This was coupled with a belief that abstinence from alcohol was dependent above all on willpower and resolve. Thus support through relapse prevention interventions was seen as unnecessary or unhelpful. A key reason that patients do not engage with alcohol treatment is that they do not feel it is needed (May *et al*. 2019, Substance Abuse and Mental Health Service Administration 2021). This finding has been echoed in previous qualitative studies of outpatients with ArLD (Mellinger *et al*. 2018, Mellinger *et al*. 2024) where it has also been recognised that fear of death is insufficient to induce alcohol abstinence (Blaxter and Cyster 1984). This study demonstrates that this finding holds true in patients with advanced liver disease, not in spite of, but because of the immediate and serious risk of harm posed by future alcohol use. For many, this generates high levels of motivation, enhances self-efficacy, and therefore creates a belief that additional support is not required.

High levels of motivation and confidence in one’s ability to remain abstinent should be highly desirable traits when considering relapse prevention. However, since we know that returning to alcohol is common (and potentially deadly) in this population, this enhanced motivation may be insufficient to maintain abstinence without additional support or may dissipate with time after discharge. Moreover, the confidence exhibited by patients might in fact represent overconfidence, and the belief that individual willpower is the key determinant of behaviour change may be misplaced (Mellinger *et al*. 2024). These traits therefore represent a double-edged sword, since they are important components of behaviour change, yet create a barrier to behaviour change intervention. Close follow-up of patients after discharge with interval assessments of levels of confidence and motivation could not only provide important insights, but also be clinically useful in helping to time and target interventions effectively.

Several behaviour change theories recognise the importance of self-efficacy as a key construct, including the Social Cognitive Theory (Bandura 1977), the Health Belief Model(Becker and Rosenstock 1974), the Theory of Planned Behaviour (Ajzen 1985), and the COM-B model (Michie *et al*. 2011). Indeed, many relapse prevention therapies have been designed to increase self-efficacy (Kruger *et al*. 2021). We found an enhanced sense of self-efficacy in our population. Theory-based behaviour change interventions for this population should perhaps therefore focus on other constructs, such as the importance of the social and physical environment. Alternatively, an intervention might be designed to sustain and support enhanced self-efficacy, rather than focus on its generation.

A small group of participants in this study identified as having severe substance dependence and described themselves as a present or past ‘alcoholic’. These patients may require more intensive support and intervention to remain abstinent. Acceptance of the label ‘alcoholic’ is a condition of participation in Alcoholics Anonymous (AA) and in this context is seen as a critical step towards recovery. Patients who take on this identity, will therefore likely benefit from referral to AA and to other 12 steps facilitation interventions, which have the advantages of being ubiquitous, effective, and essentially free (Kelly 2017, Kelly *et al*. 2020).

However, many patients in this study rejected the identity of ‘alcoholic’ and still considered that relapse prevention was only needed for ‘bad drinkers’. It may seem axiomatic that an individual with advanced ArLD has AUD, and although the term AUD describes the full spectrum of problems related to alcohol use, a more binary model that distinguishes between ‘alcoholics’ and other alcohol users has been found to be dominant in UK studies of alcohol use (Wilson *et al*. 2013, Khadjesari *et al*. 2015, Khadjesari *et al*. 2018) and remains prevalent in public discourse (Morris *et al*. 2023). Social identity theory proposes that individuals derive their sense of self from membership of groups and develop positive attitudes towards groups of which they are a member, and negative attitude towards those to which they do not belong (Tajfel and Turner 1978, Notley *et al*. 2023). In this case, the socially constructed identity is challenged by a diagnosis of advanced ArLD resulting in a defensive mechanism called ‘othering’ (Morris *et al*. 2021). Morris *et al*. have proposed that by pointing to the ‘alcoholic other’ (or ‘bad drinker’), problem drinkers maintain their current identity and avoid adopting one with more negative connotations, including a loss of control and stigma (Morris *et al*. 2022, Schomerus *et al*. 2022). Fear of this identity and its associated stigma may therefore prevent even patients with advanced ArLD from engaging with local alcohol services and with relapse prevention (Vaughn-Sandler *et al*. 2014, Schomerus *et al*. 2022).

Morris *et al*. further propose that problem recognition is an important and discreet concept for change processes amongst harmful drinkers (Morris *et al*. 2021, Morris *et al*. 2025). The patients in this study recognised that they had a ‘problem’ (ArLD) but did not equate this to problematic alcohol use or to their own definition of an ‘alcoholic’. It is possible that a specific focus on problem recognition and on interventions that are likely to enhance this would be helpful.

The findings of this study might also support a more pragmatic approach to relapse prevention. Participants identified specific triggers for heavy drinking and recognised the need to replace the role alcohol had played in their lives as well as addressing the environment in which alcohol excess had occurred. Relapse prevention should therefore provide patients with coping strategies and alternatives to alcohol, again drawing behaviour change theories and models beyond initial motivation. Environmental restructuring is an underutilised behaviour change technique that could potentially be used for this population (Wylie *et al*. 1995). These techniques focus on external, rather than internal triggers for alcohol use and therefore do not require the individual to adopt a revised social or personal identity or to engage in traditional psychological therapies.

NoLo products may have a role to play since they provide a replacement for alcohol use and might enable patients to maintain a positive alcohol identity. The danger however is that NoLo products provide the same social and environmental cues as alcohol use; they have also been linked to increased craving amongst patients with AUD (Caballeria *et al*. 2022). The views of the participants in this study reflect that mixed picture, and the place of NoLo consumption in the management of patients with ArLD is far from clear and merits further research (Marjot and Dhanda 2025).

Finally, a move towards integrated care of AUD and ArLD, hosted in the liver clinic, might help frame relapse prevention as an important and routine part of management. There is emerging evidence that this strategy can improve clinical outcomes (Elfeki *et al*. 2023, Sedarous and Flemming 2023, Dhanda *et al*. 2024). In this context, relapse prevention would be a treatment for all patients with ArLD, not only those who identify as having alcohol dependence. One might also consider routine prescription of pharmacotherapies for AUD in patients with advanced ArLD. All these measures may help reduce the stigma around relapse prevention interventions. A revised identity of a patient with liver disease might be more easily accepted that that of an ‘alcoholic’. If alcohol use is framed in the context of external factors/triggers, then framing relapse prevention as part of the medical management of liver disease keeps any ‘blame’ external as well and might allow patients to maintain their previous positive self-identity while still engaging with interventions.

### Limitations

The main limitation of this study is the potential for selection bias and the potential limitations on the generalisability of the findings. This study represents the views of patients drawn from two hospital sites in England who were all of white ethnicity and mostly born in the UK. The distribution of deprivation did not reflect the wider population of patients with ArLD cirrhosis in the UK. It was necessary for patients to speak English and be clinically well enough to participate in an interview. Approximately half of the patients approached were either too unwell or unwilling to participate in the study. The first author is trained as a physician and that may have created researcher bias in the interviews and their analysis.

## Conclusions

Following a hospital admission, patients with advanced ArLD understood the risks of returning to alcohol but were reluctant to engage in relapse prevention therapies. Instead, they expressed a renewed confidence in their ability to remain abstinent and a belief in willpower as the key determinant of long-term abstinence. The label ‘alcoholic’ was often rejected, and alcohol consumption rationalised in the context of social norms—thus relapse prevention, in participants’ views, was not needed. Understanding these views in the context of behaviour change theory and identity theory could guide the development of services and interventions, which address the identified barriers. In particular, further integration of liver care with AUD treatment, and a pragmatic approach to relapse prevention, could be considered.

## Supplementary Material

COREQ_Checklist_PRAC_Qual_A1_agaf027

## Data Availability

The data underlying this article cannot be shared publicly to protect the privacy of individuals that participated in the study.

## Notes

*Conflict of interest:* None of the authors have relevant personal or funding interests to declare. In full interests are as follows: C.O. has received research funding from the National Institute for Health and Care Research. C.O. is an employee of Cambridge University Hospitals NHS Foundation Trust. G.M. has served as a speaker for NHS Providers. G.M. has served as an advisory board member for the National Institute for Health and Care Excellence. G.M. has received research funding from the National Institute for Health and Care Research and the Health Foundation. G.M. is an employee of the University of Cambridge. T.A. has nothing to declare. M.A. has received research funding from GlaxoSmithKline. M.A. is an employee of Cambridge University Hospitals NHS Foundation Trust. Caitlin Notley is an employee of the University of East Anglia.
